# CAREGIVING AND PREPARATION FOR RETIREMENT

**DOI:** 10.1093/geroni/igz038.1401

**Published:** 2019-11-08

**Authors:** Shirley L Porterfield, Eunsun Kwon

**Affiliations:** 1 University of Missouri-St Louis, St. Louis, Missouri, United States; 2 St. Cloud State University, St Cloud, Minnesota, United States

## Abstract

Saving for retirement should begin with the first job, but preparation with respect to determining a specific retirement age and plans for post-retirement life, generally occurs closer to the retirement date. However, among those who provide care for family or close friends who are elderly and/or have disabilities, retirement preparation may take a back seat to more pressing current concerns. While we know quite a lot about patterns of saving for retirement and the factors that influence those patterns, we know little about retirement expectations and patterns of thinking about and planning for the broader retirement experience, particularly among caregivers. This paper uses data from the 2008-2016 rounds of the nationally-representative 1979 National Longitudinal Survey of Youth to examine retirement expectations and five areas of retirement preparation (reading, using a computer app, consulting a financial planner, calculating income, or attending meetings) among employed adults (ages 51-59 in 2016) who are or are not providing care for someone in or out of their household. Longitudinal analysis finds significantly lower retirement preparation among adults caring for someone inside versus outside the household, as well as significantly lower preparation activities among female versus male caregivers. Caregiving influences employment and, in turn, the types of retirement accounts held by men and women. Although caregiving is associated with decreased retirement savings among both men and women who have pension accounts, retirement preparation activities in 2008 and 2012 are associated with higher retirement savings in 2016.

